# The Advantages of FEV_1_ Percent Predicted Change During Bronchial Challenge Testing

**DOI:** 10.1007/s00408-025-00823-5

**Published:** 2025-07-05

**Authors:** James Dean, Augusta Beech, Dave Singh

**Affiliations:** 1 Division of Immunology, Immunity to infection and Respiratory Medicine, School of Biological Sciences, Faculty of Biology, Medicine and Health, The University of Manchester and Manchester University NHS Foundation Trust, Manchester, UK; 2 Medicines Evaluation Unit, Southmoor Road, Manchester, M23 9QZ UK

**Keywords:** Methacholine, Bronchial challenge, Asthma, Percent predicted, Hyperresponsiveness

## Abstract

**Background:**

The methacholine challenge requires a 20% fall in forced expiratory volume in one second (FEV_1_). The fall is measured as litre (L) change from the pre-challenge (baseline) value. A higher baseline FEV_1_ requires a greater volume change to reach a 20% fall. The aim of this study was to evaluate change using percent predicted, which may remove dependence on the baseline value.

**Methods:**

Challenge data from a cohort of 114 asthma patients was re-analysed. The dose causing an 20% fall from baseline (PD_20_) was compared to a 15% fall in predicted value (PD_15%_) for the classification of bronchial hyperresponsiveness.

**Results:**

There was significant agreement between PD_20_ and PD_15%_ (r = 0.95, *p* < 0.0001), with an ICC of 0.97. PD_20_ was significantly higher than PD_15__%_ (0.0055 mg, *p* < 0.0001). Greater decreases in FEV_1_ were observed with PD_20_ versus PD_15%_ (21.4% pred vs 19.1% pred respectively, *p* = 0.0004), with 29% of patients requiring at least one additional dose of methacholine to achieve PD_20_ compared to PD_15%_. A higher baseline FEV_1_ resulted in higher PD_20_ values, whereas no relationship was found for PD_15%_. Variability in FEV_1_ between repeated visits (n = 15) was associated with the change in PD_20_, but not the change in PD_15%_.

**Conclusion:**

We suggest a PD criteria based on 15% predicted change should be used for bronchial challenge testing. This method is less influenced by baseline airflow obstruction, and is a more efficient and safer way of measuring airway hyperresponsiveness.

**Supplementary Information:**

The online version contains supplementary material available at 10.1007/s00408-025-00823-5.

## Introduction

Increased bronchial hyperresponsiveness to inhaled challenge agents is a recognised feature of asthma. Current guidance for methacholine challenges requires a patient to receive doubling or quadrupling concentrations until their forced expiratory volume in one second (FEV_1_) has fallen 20% from the baseline value, with the dose causing an exact 20% fall (PD_20_) used to grade the severity of hyperresponsiveness [1]. A higher baseline FEV_1_ (L) requires a greater volume change to reach PD_20_ compared to a lower baseline FEV_1_ (L). FEV_1_ tends to be higher in those of greater height, younger age and in males. This may introduce demographic bias in bronchial challenge testing. There may also be variability between visits in baseline FEV_1_ resulting in a change in the volume required to meet the PD_20_ threshold. Furthermore, asthma patients with more airflow obstruction will require a smaller decrease in lung function to be classified as hyperresponsive, which may increase the proportion of positive results at lower lung function values.

Using a % change from an absolute value results in a dependence on the baseline value, where the change required will increase or decrease in line with the baseline value itself. Reporting change as a % of predicted theoretically removes this dependence, as the change is a percentage of the fixed predicted value determined by the individual’s demography. The alternative approach of assessing change in terms of percent predicted has been successfully employed in bronchodilator responsiveness testing to remove demographic bias [2, 3].

To the authors knowledge, using percent predicted to calculate FEV_1_ changes during a bronchial challenge has not been evaluated, despite the potential advantages of this method. We have therefore compared these two methods of bronchial challenge testing with regards the classification of bronchial hyperresponsiveness in patients with asthma.

## Methods

### Participants

We performed a retrospective analysis of methacholine challenge results obtained from 114 asthma patients with a physician diagnosis, participating in research studies at a single site between 2008 and 2019. Never and ex-smokers (< 1 pack year history) were included if their records included full methacholine challenge data, including spirometry after each dose of methacholine. Patients taking inhaled corticosteroid (ICS) were on a stable dose for at least 4 weeks prior to the challenge. Patients were excluded using the following criteria: known respiratory disorder other than asthma; current aortic aneurysm; recent myocardial infarction or stroke; hypertension; respiratory infection or use of antibiotics within 4 weeks; or presence of a contraindication to any the procedures, that in the investigator’s opinion made it unsafe for the patient to participate. A sub-cohort of 15 patients had repeated methacholine challenge data, and were analysed for variability. All patients provided written informed consent using protocols approved by North West—Greater Manchester East Research Ethics Committee (05/Q1402/41; 14/NW/0048; 12/NW/0632) or North Manchester Research Ethics Committee (08/H1006/54).

### Study Procedures

Spirometry was performed as per American Thoracic Society (ATS) / European Respiratory Society (ERS) guidelines [4, 5] using either the MicroLab spirometer (MicroMedical, Rochester, UK) or the NDD Easy On-PC system (NDD Medical Technologies, Zurich, Switzerland). Both devices met recognised specifications for spirometry equipment [4, 5] and their accuracy had been verified prior to patient testing. Each patient was tested using the same device throughout their visit. During post challenge time points, FEV_1_ only was measured. Predicted values were calculated using the Global Lung Function Initiative (GLI) 2012 equations [6].

Each patient complied with the ERS pre-test guidelines [1] prior to each methacholine challenge. Challenges were performed using either the 5-breath method (n = 85) as per ATS/ERS 1999 guidelines [7] or more recently using the tidal breathing method (n = 29) as per the updated ERS 2017 guidelines [1]. The 5-breath method utilised the Markos Mefar dosimeter and the MB3 nebuliser (Mefar, Brescia, Italy) to produce an output of 9µL per breath, giving a total volume dose of 45µL per concentration, giving incremental doses that doubled from 0.0006 to 0.3312 mg. Methacholine dose was calculated for this method with a fraction of respirable particles of 0.46, using the formula: concentration(mg/ml) x respirable fraction(0.46) x ml dose(0.045). The tidal breathing method utilised a 20 s dose duration [8] using the AeroEclipse BAN II nebuliser (Trudell Medical International, Ontario, Canada), with dose calculated as per ERS 2017 guidelines [1], using the formula: concentration(mg/ml) / 16 mg/ml × 0.68. This produced incremental doses that doubled from 0.0013 to 0.6840 mg.

PD_20_ was calculated as per the ERS 2017 statement [1], using interpolation between the final two methacholine administrations to find the dose resulting in a 20% fall in FEV_1_. The severity of methacholine hyperresponsiveness was categorised using the ERS 2017 statement [1], and hence participants were considered hyperresponsive if their PD_20_ was below 0.4 mg (i.e. borderline hyperresponsive). Change in percent predicted FEV_1_ was calculated based on the formula for bronchodilator responsiveness provided in the ERS/ATS interpretive strategies document [9], specifically: Change in FEV_1_% predicted = ((post-methacholine FEV_1_ − post-diluent FEV_1_) * 100) / predicted FEV_1_. This was used to calculate PD values using the same interpolation method as PD_20_. Global Lung Initiative 2012 equations [5] were used in these calculations.

### Statistical Analysis

PD data was transformed using Log^10^ + 10 for statistical analysis to account for PD values less than 1, and back transformed to report mean/median values. Normality of data was assessed using the D’Agostino & Pearson test. Comparisons were made using t-tests or Wilcoxon tests, paired/unpaired where appropriate. Correlation analysis was performed using Pearson or Spearman r test, as appropriate. Intra-subject variability and agreement between different methods were assessed using the intra-class correlation coefficient (ICC), based on a two-way mixed or two-way random model, respectively, with absolute agreement; these are interpreted as excellent (> 0.75), fair to good (0.40–0.75) or poor (< 0.40) [10]. Multiple Linear Regression was performed for the difference between PD methods as a least squares main effects model, considering baseline FEV_1_% predicted, age, height and sex. In patients with repeat challenges, the mean doubling dose (DD) difference between the two challenges was calculated in Excel (Microsoft Corporation; formula: = (INT(LN(PD2/PD1)/LN(2)) + 2^(MOD(LN(E2/D2)/LN(2),1)) − 1)), and the 95% range calculated as previously described [11, 12]. The latter gives the repeatability of the PD method based on within-subject between-measurement standard deviation, accounting for the inherent variability of both the first and second challenge. Bland Altman plots were constructed as the DD difference between repeated PD values (y-axis) versus the mean untransformed PD values (x-axis). All analysis was performed using Prism 10 (Graphpad, CA, USA) other than ICC analysis, which was performed using SPSS 25.0 (IBM, NY, USA). *p* < 0.05 was considered statistically significant.

## Results

The participants (n = 114) were predominantly male (61%), never-smokers (90%), with more than half prescribed inhaled corticosteroids. Full demographic data are shown in Table 1. Differences in challenge methodology across our cohort did not result in significantly different PD_20_ values (see supplement).

**Table 1:** Participant characteristics

Characteristic	Data (n = 114)
Sex (male/female)	69/45
Age, years	43 [31–53]
Never-smokers/ex-smokers	103/10
ICS prescribed (%)	55%
Pre BD FEV_1_% predicted	91 (± 14)
Pre BD FEV_1_/FVC ratio	0.74 (± 0.08)
PD_20_ (mg)	0.0172 [0.0070–0.0689]
Severity of hyperresponsiveness (% of cohort):	
Normal (PD_20_: > 0.4 mg)	20%
Borderline (PD_20_; 0.1–0.4 mg)	14%
Mild (PD_20_; 0.025–0.1 mg)	23%
Moderate (PD_20_; 0.006–0.025 mg)	29%
Marked (PD_20_; < 0.006 mg)	14%

Data is n, % of subjects, median [IQR] or mean (± SD) where appropriate. Data missing from subject records: smoking status = 1; ICS prescribed = 2. ICS—Inhaled corticosteroid; BD—Bronchodilator; FEV_1_—Forced expired volume in 1 s; FVC—Forced vital capacity; PD_20_— provocative dose resulting in a 20% fall in FEV_1_

### PD_20_ compared to calculations using % predicted

**Fig. 1:**
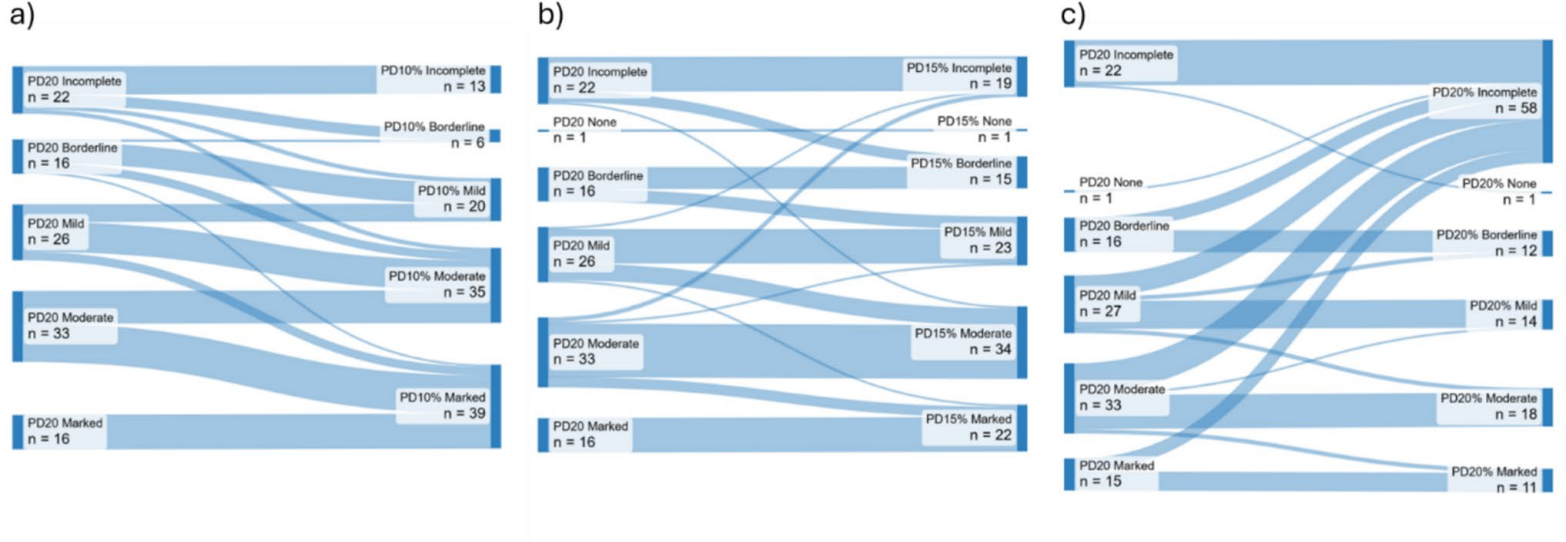
Difference in reported hyperresponsiveness compared to PD_20_ when using and **a** PD_10%_, **b** PD_15%_ and **c** PD_20%_. Incomplete — the required fall in FEV_1_ was not achieved and hence a PD value was not calculated. For PD_20_, these patients are considered to not be hyperresponsive as they did not respond by dose that equates to a positive test (i.e. > 0.4 mg). Some patients who were hyperresponsive with PD_20_ but had an incomplete PD_10%_, PD_15%_, or PD_20%_ value may become positive if further doses are administered and therefore were not fully classified using PD_%_; None — patients who had the required fall in FEV_1_, but the PD or PD_%_ value was above the threshold for a positive test (i.e. > 0.4 mg); PD_20 _— provocative dose resulting in a 20% fall in FEV_1_; PD_10% _— Provocative dose resulting in a 10% fall in the percent predicted value for FEV_1_; PD_15% _— provocative dose resulting in a 15% fall in the percent predicted value for FEV_1_; PD_20% _— provocative dose resulting in a 20% fall in the percent predicted value for FEV_1_

PD_20_ calculations showed that 91 (80%) patients were classified as hyperresponsive with a positive result at < 0.4 mg. To identify an equivalent threshold for % change in predicted FEV_1_, the number of patients meeting 10%, 15% and 20% thresholds was plotted (Fig. S1); these were termed PD_10%_, PD_15%_, and PD_20%_, respectively. In the 91 patients with a positive PD_20_ value, all patients achieved PD_10%_ and 97% achieved PD_15%_, but only 60% achieved PD_20%_. The agreement was calculated for patients who achieved both PD_20_ and % change in predicted FEV_1_ criteria; there was significant agreement between PD_20_ and PD_20%_ (r = 0.96, *p* < 0.0001; ICC = 0.98 (95% CI 0.96–0.99), *p* < 0.0001), PD_20_ and PD_15%_ (r = 0.95, *p* < 0.0001; ICC = 0.97 (95% CI 0.90–0.98), *p* < 0.0001), but less agreement between PD_20_ and PD_10%_ (r = 0.86, *p* < 0.0001; ICC = 0.81 (95% CI -0.08–0.94), *p* < 0.0001). More patients remained in the same category when using PD_15%_ (74%, Fig. 1b), compared to PD_10%_ and PD_20%_ (46% and 61% respectively, Fig. 1a, c). PD_10%_ resulted in at least half of patients (54%) becoming more hyperresponsive (Fig. 1a), whereas PD_20%_ was not achieved in an additional 37 patients (32%) compared to PD_20_ (Fig. 1c). Subsequent analysis used PD_15%_ due to this close relationship to PD_20_ results (unlike PD_10%_) and it being commonly achieved alongside PD_20_ (unlike PD_20%_).

**Fig. 2:**
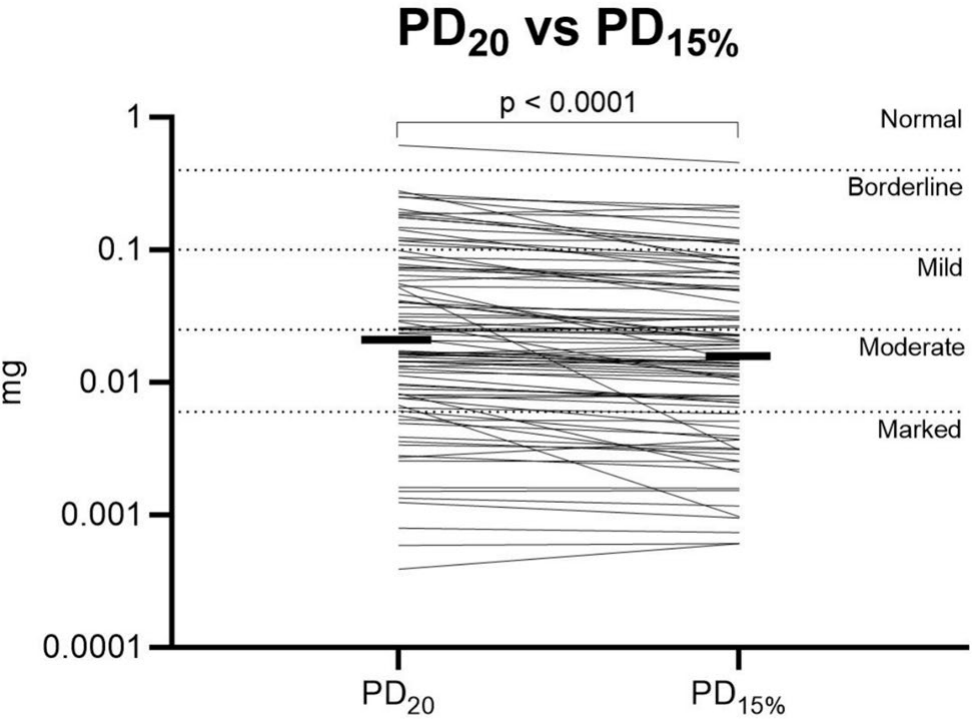
Difference in reported hyperresponsiveness when using PD_20_ and PD_15%_. Thin lines represent individual participants. Thick lines represent median value. PD_20_ versus PD_15%_ comparison included only patients with both a PD_20_ and PD_15%_ value (n = 87). PD_20 _— provocative dose resulting in a 20% fall in FEV_1_; PD_15% _— provocative dose resulting in a 15% fall in the percent predicted value for FEV_1_

In patients with a positive PD_15%_ value (n = 88), the median % fall in FEV_1_ was greater (*p* < 0.0001) for PD_20_ versus PD_15%_; 24.3% (range: 20.0–41.5%) versus 22.4% (range: 15.1–41.5%). Similarly, the median % predicted fall was greater (*p* = 0.0004) for PD_20_ versus PD_15%_; 21.4% (range: 15.4–37.5%) versus 19.1% (range: 15.1–37.5%) respectively. PD_20_ was significantly higher than PD_15%_; mean difference (95%CI) 0.0055 mg (0.0038–0.0078 mg), *p* < 0.0001 (Fig. 2). Setting the target fall in FEV_1_ using PD_15%_ resulted in 33 patients (29%) requiring at least one less dose of methacholine. Those requiring fewer doses had better baseline lung function (FEV_1_% predicted mean (95%CI) difference: 13.9% (8.7–19.2%; *p* < 0.0001; Fig. S2) and were younger (median age difference [IQR]: − 4 [− 4 to − 8] years, *p* = 0.0159), whereas height and sex were not different (both *p* > 0.05). Both PD_20_ and PD_15%_ were similar between patients taking ICS and steroid naive patients (Fig. S3).

Change in hyperresponsiveness classification when using PD_15%_ (versus PD_20_) is shown in Fig. 1b. PD_15%_ resulted in some patients being classified as more hyperresponsive (n = 20) and 6 patients changing classification from non-responsive to hyperresponsive. A PD_15%_ value could not be calculated in 3 patients (i.e. an absolute 20% fall was met without obtaining a 15% fall in predicted).

### Relationship to Baseline Lung Function

**Fig. 3:**
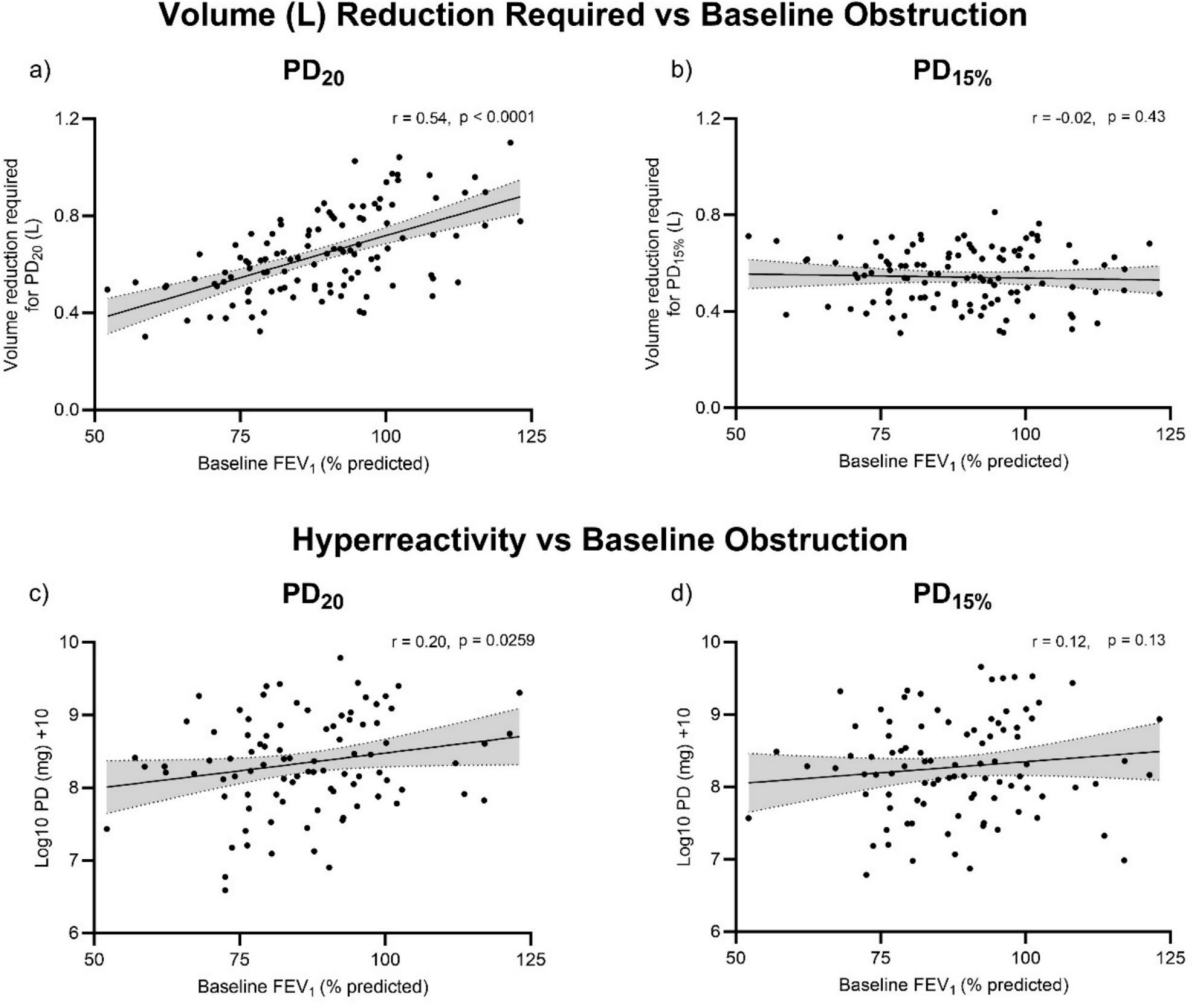
Upper panel: Relationship between baseline airflow obstruction (FEV_1_% predicted) and the volume (L) fall in FEV_1_ required to meet PD_20_ and PD_15%_ (**a**, **b**, respectively). Lower panel: Relationship between baseline airflow obstruction (FEV_1_% predicted) and hyperresponsiveness measured by log^10^ + 10 transformed PD_20_ and PD_15%_ (**c**, **d**, respectively). Correlations calculated as Spearman r value (**a**, **b**) or Pearson r value (**c**, **d**). PD_20 _— provocative dose resulting in a 20% fall in FEV_1_; PD_15% _— provocative dose resulting in a 15% fall in the percent predicted value for FEV_1_. FEV_1 _— Forced expired volume in 1 s

For the PD_20_ calculation of the required volume (L) fall in FEV_1_, there was a positive correlation between the required volume and the baseline FEV_1_% predicted (r = 0.54, *p* < 0.0001; Fig. 3a), signifying that a smaller volume fall was required in those with a greater degree of airflow obstruction at the start of the challenge. The PD_20_ observed was also associated with baseline FEV_1_% predicted (r = 0.20, *p* = 0.026, Fig. 3c). There was no correlation between the required volume (L) fall and baseline FEV_1_% predicted for the PD_15%_ criteria (r = − 0.02, *p* = 0.43; Fig. 3b), or the PD_15%_ observed and baseline FEV_1_% predicted (r = 0.12, *p* = 0.13, Fig. 3d).

**Fig. 4:**
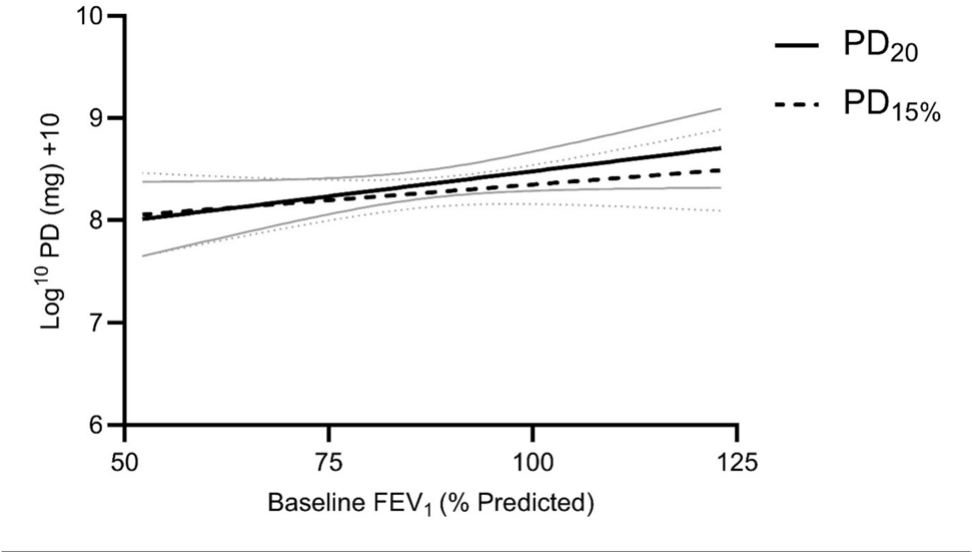
Simple linear regression (mean and error) for PD_20_ and PD_15%_ showing separation at higher baseline FEV_1_% predicted. In patients with higher baseline % predicted FEV_1_, PD_15%_ was generally lower than PD_20_ (i.e. patient labelled as more hyperresponsive). PD_20 _— provocative dose resulting in a 20% fall in FEV_1_; PD_15% _— provocative dose resulting in a 15% fall in the percent predicted value for FEV_1_. FEV_1 _— Forced expired volume in 1 s

Simple linear regression revealed a separation between PD methods at higher baseline FEV_1_% predicted values (Fig. 4), and multiple linear regression confirmed that baseline FEV_1_% predicted was a major determinant of the difference between PD_20_ and PD_15%_ (β = 0.0095, *p* < 0.0001, Table 2). Age, height and sex did not significantly predict the difference in reported hyperresponsiveness (Table 2).

**Table 2:** Multiple linear regression showing the impact of sex, age, height, and baseline airflow obstruction (FEV_1_% predicted) in predicting the change in hyperresponsiveness (PD value) when switching from PD_20_ to PD_15%_

Characteristic	PD_20_–PD_15%_		
	β Estimate	95% CI	*p*
Sex (female)	− 0.0416	− 0.1159 to − 0.0326	0.27
Age (years)	− 0.0012	− 0.0042 to 0.0018	0.42
Height (m)	− 0.0003	− 0.0017 to 0.0011	0.63
Baseline FEV_1_% predicted (%)	0.0095	0.0067–0.0123	< 0.0001

PD_20 _— Provocative dose resulting in a 20% fall in FEV_1_; PD_15% _— Provocative dose resulting in a 15% fall in the percent predicted value for FEV_1_. FEV_1 _— Forced expired volume in 1 s

### Variability

**Fig. 5:**
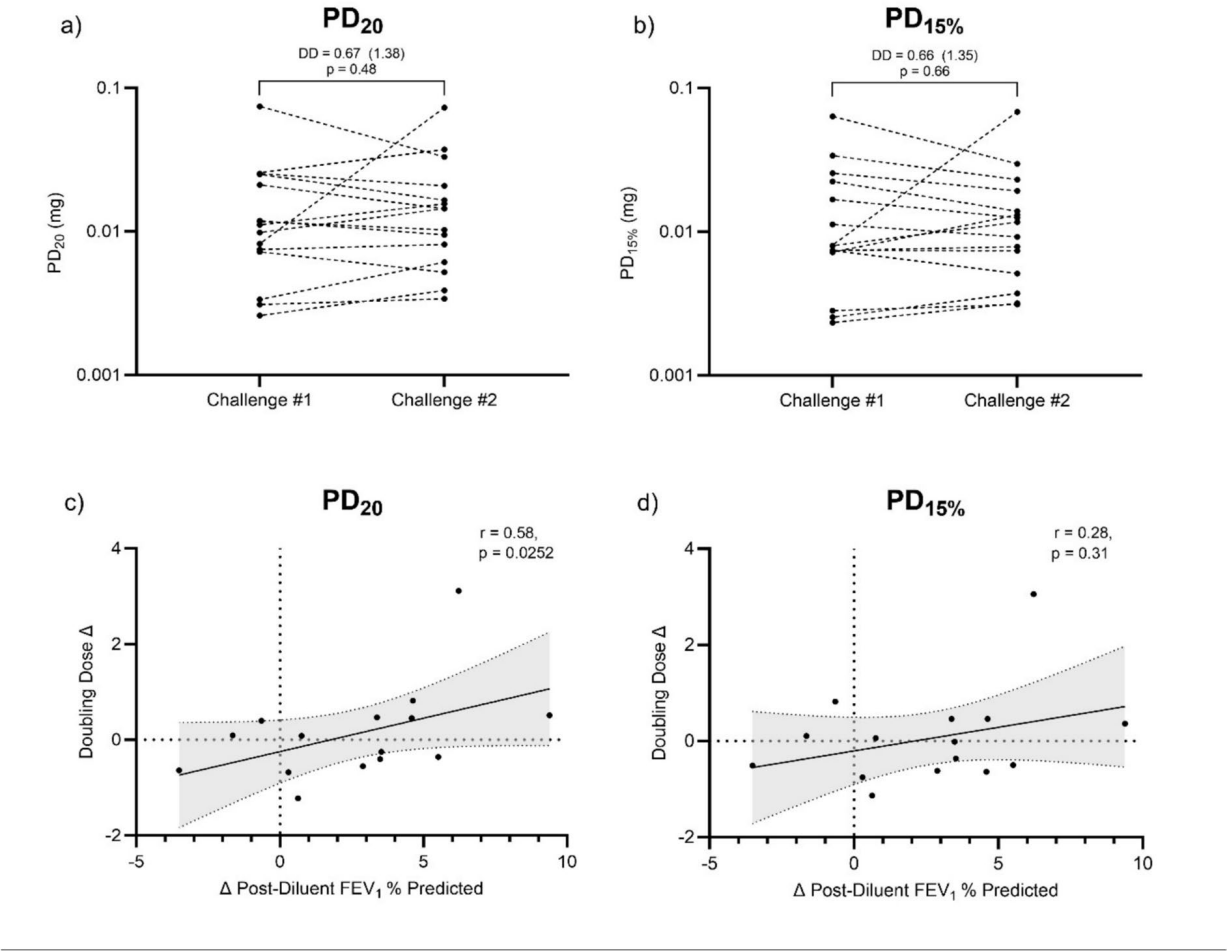
Upper panel — change in PD values between repeat visits for PD_20_ and PD_15%_ (**a**, **b**, respectively); Lower panel — relationship between the change in baseline airflow obstruction (post-diluent FEV_1_% predicted) and change in PD_20_ and PD_15%_ between repeated challenges (**c**, **d**, respectively). Correlations calculated as Spearman r value. Statistical analysis performed with log^10^ + 10 transformed data. DD — average doubling dose change (95% range for future differences); PD_20 _— provocative dose resulting in a 20% fall in FEV_1_; PD_15% _— provocative dose resulting in a 15% fall in the percent predicted value for FEV_1_. FEV_1 _— Forced expired volume in 1 s

Fifteen patients had repeat data, performed within 1 week. There was no significant change in PD value between repeated challenges for PD_20_ (mean DD difference (95% range): 0.67 (1.38), *p* = 0.48; Fig. 5a) or PD_15%_ (mean DD difference (95% range): 0.66 (1.35), *p* = 0.66; Fig. 5b). 13 out of 15 patients were within 1 DD for both PD_20 _and PD_15%_. Figure S4 shows similar variation for repeated PD_20_ values and repeated PD_15%_ values. ICC analysis showed excellent agreement between repeat visits for log transformed PD_20_ values (ICC (95% CI): 0.83 (0.49–0.94), and for log transformed PD_15%_ values (ICC (95% CI): 0.84 (0.53–0.95). A change in baseline FEV_1_% predicted between the first and second challenge correlated with the DD change in PD_20_ (r = 0.58, *p* = 0.03; Fig. 5c), while no association was seen for DD change in PD_15%_ (r = 0.28, *p* = 0.31; Fig. 5d).

## Discussion

We compared % change using absolute baseline FEV_1_ values (PD_20_) versus change in FEV_1_% predicted during methacholine challenge testing. The change in FEV_1_% predicted was less influenced by baseline FEV_1_, so was less prone to differences in baseline airflow obstruction. This method required less methacholine doses in some patients, which is safer, and may be more consistent in patients who experience greater FEV_1_ variability between visits. Reporting results as a percent of predicted is common practice across lung function testing, for example in spirometry testing and response to bronchodilators. The results reported here indicate advantages for using % predicted for methacholine challenge testing.

In bronchial challenges, a bias towards a positive result has been reported in asthma patients with greater baseline airflow obstruction, whereas no bias existed for age or sex [13]. Likewise, smokers and ex-smokers with greater baseline airflow obstruction were more likely to be classed as hyperresponsive [14, 15]. One explanation for these findings is that the PD_20_ calculation method requires a smaller volume fall in individuals with lower FEV_1_ at the start of the challenge for a positive challenge; consequently, we observed that lower methacholine doses reached PD_20_ in individuals with lower baseline FEV_1_. In contrast, there was no association between PD_15%_ and baseline FEV_1_% predicted. Using PD_15%_ therefore removes the potential for the mathematical calculation to bias the results towards hyperresponsiveness at lower lung function levels. Patients with more severe asthma are more likely to have increased bronchial hyperresponsiveness and lower lung function, but these may be independent processes as bronchial hyperresponsiveness can exist in patients with normal lung function [16]. PD_15%_ allows hyperresponsiveness to be assessed independently of the level of airflow obstruction, unlike PD_20._

While PD_15%_ was highly associated with PD_20_ in our cohort, higher doses of methacholine were used to obtain a positive result with PD_20_. A larger volume fall was required for PD_20_ compared to PD_15%_, which was more common in patients with higher baseline FEV_1_. This explains the higher doses administered to reach PD_20_ compared to PD_15%_. Methacholine challenges are generally well tolerated, with a low likelihood of severe reactions [7] or prolonged symptoms [17]. However, larger doses still increase the risk of irritable symptoms [18], severe falls in FEV_1_ [19, 20], and lead to a longer test duration. The fewer doses required to reach PD_15%_ resulted in a significantly smaller drop in lung function and a shorter challenge duration. We propose that PD_15%_ is a more efficient and safer challenge methodology.

PD_15%_ increased the reported methacholine responsiveness classification in some patients, predominantly affecting individuals with better baseline lung function, most likely due to these individuals requiring a smaller volume change for PD_15%_ compared to PD_20_. The thresholds for the severity were derived using symptoms [13], lung function variability [21], and therapy requirements [22, 23]. While the present study cannot determine the clinical impact of greater reported bronchial hyperresponsiveness with PD_15%_, it should be noted that the majority of individuals (65 out of 88 patients with a calculated PD_15%_: 73.8%) did not change classification. Alternative PD_%_ thresholds of 10% or 20% were also considered, however we found these resulted in higher reported levels of hyperresponsiveness compared to PD_20_ (i.e. PD_10%_) or resulted in incomplete challenges (i.e. % predicted fall not achieved; PD_20%_). PD_15%_ showed more consistency with current methods, and therefore the published grading system currently in use for PD_20_ is also likely to be suitable for PD_15%_.

Our data showed that both PD_20_ and PD_15%_ were repeatable with most patients having values within 1 DD between visits. A change in baseline lung function was associated with a change in PD_20_ but not PD_15%_. These results further highlight the influence that the level of baseline airflow obstruction has on the measurement of bronchial hyperresponsiveness when using PD_20_ (but not PD_15%_) due to mathematical calculation rather underlying airway pathophysiology.

Our study had some limitations. First, our analysis was retrospective in nature, such that a PD_15%_ value was not available in all patients (not calculated in 3 out of 91). A prospective study would allow continued dosing to reach a 15% fall in predicted in all patients, and also allow comparisons with healthy individuals without hyperresponsiveness to determine suitable PD_15%_ thresholds for a positive test. Second, the data included used one of two methods, with challenges performed prior to 2019 using the 5 breath method described in the ATS 1999 guidelines [7]. This method is no longer supported due to the bronchoprotective effect of deep inhalations [1, 24], possibly leading to an underestimation of hyperresponsiveness in some patients. However, given our comparison was between two calculation methods within a single challenge (PD_20_ vs PD_15%_) rather than between challenges, our conclusions are still valid. Finally, a significant portion of patients were taking ICS at the time of testing, which could suppress hyperresponsiveness to methacholine. However, the same challenge data was used to compare both methods ensuring that there was no confounding effect of ICS use.

Our results support the potential use of a PD criteria based on 15% predicted change for bronchial challenge testing. This method is less influenced by baseline airflow obstruction, and is a more efficient and safer way of measuring airway hyperresponsiveness. We encourage more research in larger cohorts to validate our findings.

## Supplementary Information

Below is the link to the electronic supplementary material.

Supplementary file1 (DOCX 253 KB)

## Data Availability

The datasets used and/or analysed during the current study are available from the corresponding author on reasonable request.

## Abbreviations

FEV_1_: Forced expiratory volume in 1 s
PD_20_: Provocative dose resulting in a 20% fall in FEV_1_
PD_15%_: Provocative dose resulting in a 15% predicted fall in FEV_1_
ICS: Inhaled corticosteroid
GLI: Global Lung Initiative
ATS: American Thoracic Society
ERS: European Respiratory Society
DD: Doubling dose
ICC: Intra-class correlation coefficient
NIHR: National Institute for Health Research
BRC: Biomedical Research Centre
FVC: Forced vital capacity
SD: Standard deviation
IQR: Interquartile range
CI: Confidence interval
