# Supplementary material for: The Advantages of FEV1 Percent Predicted Change During Bronchial Challenge Testing
Source: Lung. 2025 Jul 5;203(1):73. doi: 10.1007/s00408-025-00823-5 (PMC12227454; doi:10.1007/s00408-025-00823-5)
Supplement: Supplementary file 1 — Supplementary file1 (DOCX 253 KB) [file 408_2025_823_MOESM1_ESM.docx]

**Supplement**

The advantages of FEV_1_ percent predicted change during bronchial challenge testing.

James Dean, Augusta Beech and Dave Singh.

Effect of challenge methodology

Our study utilised historic methacholine challenge data between 2008 and 2024 conducted at a single centre. In 2016 the centre updated their spirometry equipment from turbine sensors to ultrasonic sensors. In 2019 a tidal breathing challenge methodology was adopted, whereas previously the 5 breath method was utilised. Data utilised in the study covered two time frames, 2008-2015 and 2019-2024. Hence data was collected with only two challenge-spirometer combinations: 5 Breath method with a turbine (referred to as 5B) or tidal breathing with a ultrasonic sensor (referred to as tidal).

PD_20_ in those who used the tidal method was similar to those who used the 5B method (p = 0.27). Likewise, PD_15%_ was similar between these two groups (p = 0.10). For the 5B method, using PD_15%_ resulted in 16% of patients being classified as more hyperresponsive and 4% patients changing classification from non-responsive to hyperresponsive. For the tidal method, using PD_15%_ resulted in 21% of patients being classified as more hyperresponsive and 4% patients changing classification from non-responsive to hyperresponsive.

Considering this and our study design (within challenge comparison of PD_20_ and PD_15%_), challenge methodology would not have impacted our results.

Supplemental Figures


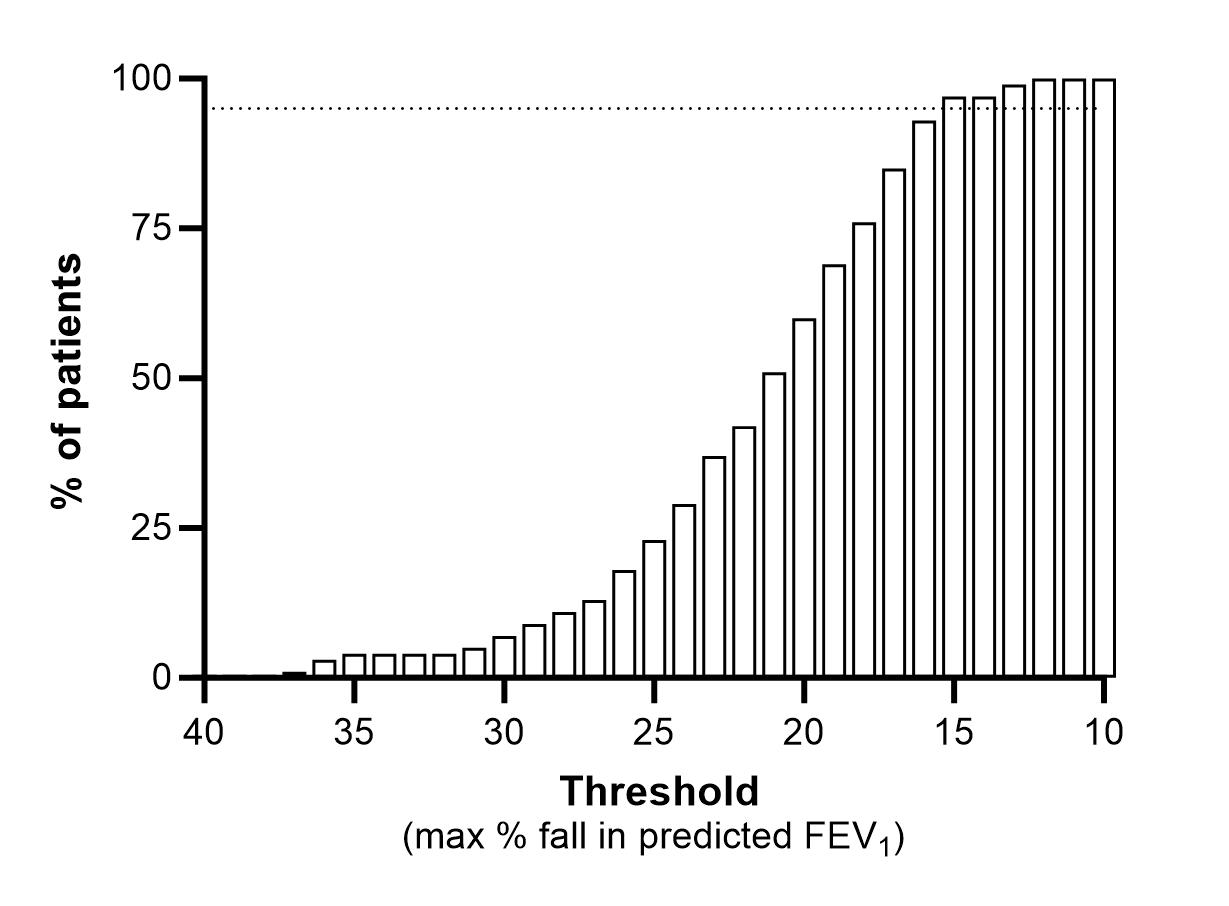


**Fig. S1** Percent of patients with a positive PD_20_ (n=91) meeting various thresholds for % predicted fall in FEV_1_ during the challenge (i.e. max % pred fall). Dotted line indicates where 95% of patients meet the threshold.

**Fig. S2** Comparison of patient characteristics between those who required fewer doses with PD_15%_ and those who did not (i.e. same/more doses), for A) baseline FEV1 % predicted (mean) and B) Age (median). FEV_1_ – Forced expired volume in 1 second

**Fig. S3** Comparison of a) PD_20_ and b) PD_15%_ between those taking ICS treatment (ICS+) and steroid naïve patients (ICS-). PD_20_ – Provocative dose resulting in a 20% fall in FEV_1_; PD_15%_ – Provocative dose resulting in a 15% predicted fall in FEV_1_

**Fig. S4** Bland Altman plots of agreement for repeated A) PD_20_ and B) PD_15%_. Solid line = line of identity; Bold dashed line = bias; Dotted lines = ±95% level of agreement. PD_20_ – Provocative dose resulting in a 20% fall in FEV_1_; PD_15%_ – Provocative dose resulting in a 15% predicted fall in FEV_1_
